# Thirty-Day Readmissions After Hospitalization for Psoriatic Arthritis

**DOI:** 10.7759/cureus.60445

**Published:** 2024-05-16

**Authors:** Fidelis Uwumiro, Solomon O Anighoro, Adetayo Ajiboye, Chukwunonso C Ndulue, God-dowell O Odukudu, Emeka S Obi, Stanley C Ndugba, Claire A Ewelugo, Evaristus Asobara, Okonkwo Ogochukwu

**Affiliations:** 1 Internal Medicine, Our Lady of Apostles Hospital, Akwanga, NGA; 2 General Practice, St. Helens and Knowsley Teaching Hospitals NHS Trust, Whiston Prescot, GBR; 3 Dermatology, Central and North West London NHS Foundation Trust, London, GBR; 4 Internal Medicine, College of Medicine, University of Lagos, Lagos, NGA; 5 Internal Medicine, Delta State University, Abraka, NGA; 6 Department of Healthcare Administration, College of Public Health, East Tennessee State University, Johnson City, USA; 7 Internal Medicine, Federal University Teaching Hospital, Owerri, NGA; 8 Internal Medicine, Nnamdi Azikiwe University, Awka, NGA; 9 Internal Medicine, College of Medicine, Ambrose Alli University, Ekpoma, NGA

**Keywords:** psoriatic arthropathy, arthropathic psoriasis, arthritis psoriatica, nationwide readmissions, thirty-day readmissions

## Abstract

Background

Psoriatic arthritis (PsA) is correlated with higher rates of major adverse cardiovascular events and autoimmune disorders than the general population, leading to more frequent hospitalizations. This study assessed the rates and characteristics of index and 30-day readmissions among adults hospitalized for PsA and evaluated the indications and predictors of 30-day readmissions across the United States.

Methodology

We analyzed the 2020 Nationwide Readmissions Database for adult PsA hospitalizations using the International Classification of Diseases, Tenth Revision, Clinical Modification codes. To compare baseline characteristics between index admissions and readmissions, we used chi-square tests. We used ranking commands to identify the most common indications for readmissions and multivariable Cox regression analysis to identify the predictors of readmissions. The primary endpoints were the rates and characteristics of index and 30-day readmissions. The secondary endpoint was the predictors of readmission within 30 days of index hospital discharge.

Results

Approximately 842 index hospitalizations for PsA were analyzed. Of these, 244 (29%) resulted in 30-day readmissions, with the primary causes being acute kidney failure, major depression, and heart failure. Readmitted patients had a mean age of 48.2 years (SD = 6.4 years) compared with 54.6 years (SD = 2.2 years) in index hospitalizations (p = 0.147). More readmitted patients were uninsured than index hospitalizations (18.6% vs. 4.4%; p = 0.015). The mean length of stay for readmissions was 7.2 days compared with 3.9 days for index admissions. The mean total hospital costs were US$31,424 for index admissions and US$60,147 for readmissions (p < 0.001). Significant differences in comorbidities such as hypertension (24.8% vs. 40.1%, p = 0.032), liver disease (29% vs. 7.9%, p = 0.020), uveitis (9.4% vs. 4.5%, p < 0.001), inflammatory bowel disease (8.6% vs. 3.8%, p < 0.001), and alcohol use disorder (29% vs. 7.8%, p = 0.002) were observed between readmissions and index admissions. Age <40 years (adjusted hazard ratio (AHR) = 2.35; p = 0.047), home healthcare (AHR = 5.87; p = 0.035), residence in the same state as the hospital (AHR = 1.24; p = 0.018), and secondary diagnoses of inflammatory bowel disease (AHR = 2.33; p < 0.001) or deep venous thrombosis (AHR = 3.80; p = 0.007) were correlated with an increased likelihood of readmission.

Conclusions

About one in three hospitalizations for PsA result in readmission within 30 days of initial discharge. Age <40 years, discharge to home healthcare, and a secondary diagnosis of inflammatory bowel disease or deep venous thrombosis were correlated with an increased likelihood of readmission.

## Introduction

Psoriatic arthritis (PsA) is a chronic, progressive, and highly heterogeneous inflammatory arthropathy reported to affect between 6% and 41% of patients with psoriasis, with an equal male-to-female preponderance [1,2]. Recent epidemiological studies have highlighted the significant burden of PsA. Notably, emerging evidence points to higher hospitalization rates, comorbidity burden, and resource use among individuals afflicted by this complex syndrome [3,4].

Hospitalization, often a marker of disease severity and uncontrolled symptoms, carries substantial economic and personal costs for healthcare systems and patients. Recent evidence suggests that patients readmitted within 30 days have approximately 50% higher one-year costs than those not readmitted [5]. These readmissions, while emblematic of the chronicity and complexity of PsA, are also indicative of potential gaps in the continuum of care, warranting closer examination. Although joint involvement has been widely reported in hospitalizations for psoriasis [6], little is known about hospitalization patterns and readmissions for PsA. Hospitalizations among patients with PsA have been linked to other multisystemic causes and complications rather than PsA itself, with sepsis emerging as the single most common reason for hospitalization in recent literature [7].

Our objectives were to describe the rates and characteristics of PsA hospitalizations and identify the most common indications and predictors of 30-day readmissions.

## Materials and methods

Data source

We analyzed the 2020 Nationwide Readmissions Database (NRD), which is sponsored by the U.S. Agency for Health Care Research and Quality (AHRQ) through the Healthcare Costs and Utilization Project (HCUP). The NRD is the most extensive publicly available database for readmissions in the United States. The 2020 NRD records data from approximately 17 million hospital discharges in its unweighted form. When weighted, it provides estimates for approximately 32 million discharges. The dataset for 2020 uses the International Classification of Diseases, Tenth Revision, Clinical Modification (ICD-10-CM) for coding purposes. Importantly, it permits the recording of up to 40 discharge diagnoses for each hospitalization. These diagnoses are categorized into the following two distinct groups: principal diagnoses, which represent the primary ICD-10 code associated with hospitalization, and secondary diagnoses, which encompass all other ICD-10 codes. The NRD is an invaluable resource that offers a nationwide hospital readmission perspective. Detailed information and access to the NRD are available at https://hcup-us.ahrq.gov/nrdoverview.jsp [8]. The description of the NRD aligns with that of a previous study [9].

Ethical considerations

The NRD is classified as a limited dataset. Because of the complete deidentification and public accessibility of patient data within the NRD, institutional review board (IRB) approval was not required for this study.

Data availability

The NRD database is accessible online through the HCUP central distributor or by email request to AHRQ at hcup@ahrq.gov.

Selection criteria and outcomes

Using methods recommended by the Centers for Medicare and Medicaid Services (CMS), we identified all index hospitalizations for PsA and readmissions occurring within 30 days of initial discharge. These index events were defined as hospitalizations occurring between January 1 and November 30, 2020, where the primary diagnosis was PsA (ICD-10 codes: L40.50, L40.51, L40.52, L40.53, and L40.59). We specifically chose this period to allow for a 30-day follow-up window for each patient hospitalized within the same calendar year. This choice was necessary because of the limitations of the NRD, which is published annually and only allows for the linkage of hospitalizations within the same calendar year. Unfortunately, the NRD does not permit the linkage or follow-up of patient information across different calendar years. For readmissions, we excluded index events in which the patient died in the hospital because there was no risk of readmission and patients with complicated comorbidities such as cancer or immunosuppression because these conditions would greatly increase the risk of readmission. We used the ICD-10-CM diagnostic codes to identify all secondary diagnoses, comorbidities, and complications. Two authors independently reviewed all ICD-10-CM codes to confirm their validity, achieving a discrepancy in the coding of <2%. When a mutual agreement on coding could not be achieved by both authors, a third author was consulted for decisive clarification. The methodology adopted in our study meticulously aligns with prior research using NRD [10,11]. To enhance the credibility and generalizability of our research findings, we diligently adhered to the study design guidelines outlined by Khera et al. (2017) to prevent common study design errors [12].

The primary objectives of this study were to determine the rates and characteristics of index hospitalizations and readmissions for PsA. The secondary objectives were to identify the indications and predictors of 30-day readmission.

Statistical analysis

Statistical analysis was performed using Stata (StataCorp LLC, College Station, TX, USA). We used the time-to-readmission and index admission length-of-stay variables to calculate the number of days to readmission. We calculated the index hospitalization rates and 30-day readmissions for all index hospitalizations associated with PsA using the weighted NRD sample to achieve national estimates stipulated by the AHRQ [13]. To ensure that we used only population-representative data, we accounted for clustering (hosp_nrd), weighting (trendwt), and stratification (nrd_stratum) within the NRD. The normality of data distribution was assessed using the Kolmogorov-Smirnov test. Missing variables were assessed using the “mdesc” command in Stata.

A comparative analysis was conducted on baseline characteristics, inpatient mortality, hospital length of stay (LOS), total hospital charges (THCs), and other categorical variables between readmissions and index hospitalizations. Categorical variables were expressed as percentages and analyzed using Pearson’s chi-square tests. Continuous variables were summarized as means and standard deviations and compared using linear regression. We quantified the comorbidity burden using Sundararajan’s adaptation of Deyo’s Charlson Comorbidity Index (CCI) scores. This adaptation categorizes CCI into four discrete groups, each signaling an increasing risk of mortality. Specifically, a CCI score exceeding 3 is associated with an estimated 10-year mortality rate of approximately 25%. In contrast, scores of 2 and 1 are associated with lower 10-year mortality rates of approximately 10% and 4%, respectively [14,15]. We adjusted for illness severity using the all-patient refined-diagnosis-related groups’ illness severity classification, which has been reported to estimate severity and predict in-hospital mortality better than the most widely used mortality indices [16].

In addition, we explored the factors predictive of readmission using stepwise multivariable Cox regression. First, we calculated the unadjusted hazard ratios (HRs) for 30-day readmissions using univariate Cox regression analysis, considering the patient and hospital-level variables and comorbidities. Variables with p-values below 0.2 in the univariable analysis were subsequently integrated into a multivariable Cox regression model. This significance level was chosen to avoid the inclusion of variables that are only marginally related but do not significantly impact the odds of readmission in the final model. Multicollinearity was assessed using the variance inflation factor (VIF). Variables with a VIF greater than 5 were excluded from the final regression model. Statistical significance was established at p-values <0.05. The outcomes of the final regression model were summarized and reported as adjusted hazard ratios (AHRs) with 95% confidence intervals (CIs).

## Results

The mean level of missing data per hospitalization was 0.7%. Because of the minimal amount of missing data, a complete case analysis was performed.

We analyzed 842 adult hospitalizations for a primary diagnosis of PsA. All 842 patients were discharged alive, and 244 readmissions (29% of index hospitalizations) were recorded within 30 days. The most common reasons for readmission were acute PsA exacerbation (45 out of 244 readmissions; 18.4%), acute kidney failure (37 readmissions; 15.1%), major depressive disorder, recurrent and severe psychotic symptoms (37 readmissions; 15%), acute diastolic (congestive) heart failure (29 cases; 11.8%), calculus of the gallbladder and bile duct, with acute cholecystitis and obstruction (21 cases; 8.6%), other psoriasis (21 readmissions; 8.5%), and chronic peripheral venous insufficiency (17 readmissions; 6.8%).

No significant difference was observed in the mean ages of readmitted patients compared with index hospitalizations (48.2 vs. 54.6 years; p = 0.147). The comorbidity burden was higher for readmissions, with up to 44.4% of readmissions having a CCI score ≥3 compared with 20.8% of index admissions (p < 0.0001). Patients with private insurance accounted for 18.2% of readmissions compared with 25.3% for index admissions. More readmissions were uninsured than index hospitalizations (18.6% vs. 4.4%; p = 0.015). The mean LOS for index hospitalizations was 3.9 days, whereas that for readmissions was 7.2 days (95% CI = 2.32-8.44 days; p < 0.0001). On average, readmissions related to PsA lasted three days longer than index hospitalizations (Table 1). Table 1 presents other baseline characteristics and outcome differences between index hospitalizations and readmissions.

**Table 1:** Comparison of baseline characteristics of index hospitalizations and 30-day readmissions. Data are presented as total count (n) with percentages (%) unless indicated otherwise. AMA = against medical advice; MI = myocardial infarction; PCI = percutaneous coronary intervention; CABG = coronary artery bypass graft; Median household income = median household income for patient’s zip code; CCI = Charlson Comorbidity Index; CHF = congestive heart failure; CKD = chronic kidney disease; PVD = peripheral vascular disease; LOF = loss of function; GERD = gastroesophageal reflux disease

Variables	Index admissions (N = 842)	30-day readmissions (n = 244)	P-value
Mean age, year	54.6	48.2	0.147
Female	651 (77.3)	55 (22.7)	0.078
Mean LOS, days	3.9	7.2	<0.0001
Mean THC, US$	31,424	60,147	<0.0001
Aggregate hospital costs, million US$	71.67	11.06	<0.0001
Insurance status			0.015
Medicare	404 (48.0)	83 (33.9)	
Medicaid	166 (19.7)	71 (29.3)	
Private	213 (25.3)	44 (18.2)	
Self-pay	37 (4.4)	45 (18.6)	
CCI			<0.0001
0	284 (33.8)	61 (25.0)	
1	291 (34.6)	55 (22.7)	
2	91 (10.8)	19 (7.9)	
≥3	175 (20.8)	108 (44.4)	
DRG severity class			0.008
Minor LOF	216 (25.7)	42 (17.1)	
Moderate LOF	418 (49.7)	37 (15.0)	
Major LOF	188 (22.3)	166 (67.9)	
Extreme LOF	19 (2.3)	0 (0.0)	
Admission day is a weekend	208 (24.7)	56 (23.0)	0.508
Median household income (quartile)			
First (0–25th)	248 (29.4)	55 (22.4)	0.578
Second (26th–50th)	239 (28.4)	82 (33.8)	0.707
Third (51st–75^th^)	176 (20.9)	18 (7.7)	0.255
Fourth (76th–100th)	174 (20.7)	62 (25.6)	0.699
Metropolitan hospital	810 (96.2)	243 (99.8)	0.565
Teaching hospital	688 (81.7)	182 (74.7)	0.563
Hospital bed size			0.258
Small	171 (20.3)	0 (0.0)	
Medium	236 (28.1)	78 (31.9)	
Large	435 (51.7)	166 (68.1)	
AMA	5 (0.6)	0 (0.0)	0.788
Discharge quarter			0.932
First	206 (24.5)	56 (22.9)	
Second	280 (33.2)	90 (36.7)	
Third	175 (20.8)	35 (14.5)	
Fourth	180 (21.4)	63 (25.8)	
Hospital ownership			0.474
Government	101 (12.0)	16 (6.6)	
Private, not-for-profit	682 (81.0)	192 (78.6)	
Private, investor-owned	6 (7.0)	36 (14.8)	
Resident of the same state as the hospital	811 (96.3)	243 (99.8)	0.530
Dyslipidemia	173 (20.6)	64 (26.3)	0.048
Old MI	12 (1.4)	1 (0.5)	0.703
Old PCI	3 (4.0)	3 (1.1)	0.526
Old CABG	24 (2.8)	26 (10.5)	0.217
Atrial fibrillation	28 (3.3)	5 (2.2)	0.616
Psoriasis vulgaris	73 (8.7)	45 (18.6)	0.371
COPD	63 (7.5)	26 (10.5)	0.738
Old stroke	40 (4.8)	0 (0.0)	0.500
Acute gout	61 (7.2)	26 (10.5)	0.679
Hypertension	338 (40.1)	61 (24.8)	0.032
DM types 1 and 2	141 (16.8)	19 (7.9)	0.423
Obesity	85 (10.1)	54 (22.0)	0.163
CHF	69 (8.2)	64 (26.2)	0.055
CKD	125 (14.8)	64 (26.1)	0.302
Liver disease	67 (7.9)	71 (29.0)	0.020
Smoking	5 (0.6)	8 (3.1)	0.788
Anemia	43 (5.2)	45 (18.4)	0.078
Alcohol use disorder	66 (7.8)	71 (29.0)	0.021
Depression	176 (20.9)	37 (15.3)	0.652
Deep venous thrombosis	27 (3.2)	3 (1.2)	0.719

Thirty-day readmissions resulted in a total of 1,892 hospital days. The mean THCs were US$31,424 for index hospitalizations versus US$60,147 for readmissions (Table 1). Readmissions had US$28,723 (p < 0.0001) higher mean THC than index hospitalizations. The aggregate THC for 30-day readmissions was US$11.06 million (Table 1). Significant differences were observed in the prevalence of hypertension (24.8% vs. 40.1%; p = 0.032), liver disease (29% vs. 7.9%; p = 0.020), alcohol abuse (29% vs. 7.8%; p = 0.002), inflammatory bowel disease (8.6% vs. 3.8%; p < 0.0001), and uveitis (9.4% vs. 4.5%; p < 0.0001), between PsA readmissions and index admissions (Table 1).

In the multivariable logistic regression analysis, age <40 years (AHR = 2.35; p = 0.047), discharge to HHC (AHR = 5.87; p = 0.035), residence in the same state as the hospital (AHR = 1.24; p = 0.018), as well as a secondary diagnosis of inflammatory bowel disease (AHR = 2.33; p < 0.001) or deep venous thrombosis (AHR = 3.80; p = 0.007) were significantly associated with an increased likelihood of readmission (Table 2).

**Table 2:** Univariate and multivariate Cox regression models of predictors of 30-day readmissions for psoriatic arthritis. Columns are presented as adjusted hazards ratio with corresponding p-values. Only covariates with a significance level of p < 0.2 on univariate Cox regression analysis were added to the multivariable Cox regression. Median annual income quartiles are divided as follows: first, $1-$49,999; second, $50,000-$64,999; third, $65,000-$85,999; fourth, $86,000 and above. HR = hazards ratio; LOF = loss of function; MI = myocardial infarction; PCI = percutaneous coronary intervention; CABG = coronary artery bypass graft; Median household income = median household income for patient’s zip code; LOF = loss of function; SLE = systemic lupus erythematosus; APR-DRG = all-patient refined-diagnosis-related groups

Variables	Univariate Cox regression		Multivariate Cox regression	
	Unadjusted HR	P-value	Adjusted HR	P-value
Female	0.32	0.100	0.56	0.675
Age, year				
≥18 and <40	2.77	0.171	2.35	0.047
≥40 and <60	1.08	0.914		
≥60	0.36	0.205		
Number of procedures				
0–1	0.83	0.803		
≥2	1.21	0.803		
Discharge disposition				
Routine discharge	0.61	0.396		
Discharge to other facilities	0.44	0.442		
Discharge to home healthcare	5.02	0.014	5.87	0.035
Discharge quarter				
First quarter	Reference	Reference		
Second quarter	1.12	0.892		
Third quarter	0.71	0.719		
Fourth quarter	1.13	0.882		
Hospital control				
Government	Reference	Reference		
Private, not-for-profit	1.73	0.617		
Private, investor-owned	4.09	0.268		
Insurance status				
Medicare	Reference	Reference		
Medicaid	2.09	0.237		
Private including HMO	0.92	0.917		
Self-pay	5.52	0.064	12.3	0.154
Charlson Comorbidity Index				
0	Reference	Reference		
1	2.26	0.363		
2	1.07	0.959		
≥3	1.30	0.028		
APR-DRG severity class				
Minor LOF	Reference	Reference	Reference	Reference
Moderate LOF	0.89	0.873		
Major LOF	0.25	0.039	1.29	0.161
Median household income (quartiles)				
First (0–25th)	Reference	Reference		
Second (26th–50th)	1.56	0.540		
Third (51st–75^th^)	0.48	0.530		
Fourth (76th–100th)	1.62	0.514		
Teaching hospital	1.28	0.760		
Admitted on a weekend	0.26	0.199	0.63	0.026
Hospital bed size				
Small	Reference	Reference		
Medium	2.73	0.360		
Large	2.90	0.334		
Discharge quarter				
First	Reference	Reference		
Second	1.01	0.969		
Third	1.04	0.758		
Fourth	0.94	0.675		
Hospital ownership				
Government	Reference	Reference		
Private, not-for-profit	0.94	0.688		
Private, investor-owned	1.20	0.353		
Length of hospital stay, day				
1–2	2.48	0.164	6.73	0.090
3–5	0.19	0.025	1.71	0.637
≥6	1.96	0.260		
Total hospital charge	1.04	0.052	0.99	0.966
A resident of the same state as the hospital	1.03	0.121	1.24	0.018
Dyslipidemia	0.32	0.254		
Old MI	0.05	<0.0001	1.33	0.332
Old PCI	1.60	<0.0001		
Old CABG	3.43	0.274		
Atrial fibrillation	1.62	<0.0001	1.30	0.196
Psoriasis vulgaris	5.63	0.010		
COPD	1.03	0.976		
Old stroke	0.58	<0.001	1.12	0.616
Acute gout	1.63	0.652		
Hypertension	0.66	0.491		
DM type 1 and 2	1.84	0.398		
Obesity	0.76	0.782		
CHF	4.32	0.053	1.32	0.201
CKD	1.31	<0.0001	1.47	0.090
Liver disease	4.33	0.045	1.42	0.056
Smoking	1.24	0.347		
Anemia	1.48	0.723		
Alcohol use disorder	2.64	0.295		
Depression	0.77	0.760		
Deep venous thrombosis	7.35	0.047	3.80	0.007

## Discussion

The index analysis of the nationwide inpatient sample provided several insights. At the outset, the observation that 17.2% of all hospitalizations resulted in a readmission within 30 days highlights the inherent complexities of PsA management. This frequency indicates that there is either an inherent challenge in treating PsA, post-discharge complications, or that other concomitant health conditions might influence the need for subsequent hospitalizations. While it is intuitive to associate PsA as a leading cause for readmission, the presence of severe health conditions, ranging from acute kidney failure and major depressive disorder to congestive heart failure, amidst the reasons for readmission becomes alarming. This spectrum of conditions indicates that patients diagnosed with PsA may be predisposed to a cascade of other health complications. Such a predisposition may be attributed to the systemic nature of the disease, the impact of chronic inflammation, or the adverse effects of the treatments administered.

The lower mean age (48.2 years) of readmitted patients, in contrast to index hospitalizations (54.6 years), provides a noteworthy perspective. One might speculate that younger patients due to occupational, societal, or personal commitments may be more prone to noncompliance or inadequate post-hospitalization care, leading to readmissions. However, this age differential was not statistically significant; hence, caution should be exercised before drawing concrete inferences. A distinct disparity was observed in the comorbidity burden between the index and readmission groups. The finding that 44.4% of readmissions had a CCI score ≥3, almost double that of index admissions, accentuates the necessity for comprehensive patient evaluation and meticulous post-discharge follow-ups, especially focusing on potential comorbid conditions. The economic implications of PsA hospitalizations are equally profound. The extended LOS during readmissions, almost doubling that of initial hospitalizations, together with the significantly elevated hospital charges accentuate the financial burden on the healthcare system. Furthermore, with a large percentage of readmissions being uninsured, there is an unmistakable strain on hospital resources and the broader health economic fabric. Additionally, the higher prevalence of conditions such as hypertension, liver disease, and alcohol use disorder in the PsA readmission cohort vis-à-vis index admissions begs a deeper understanding of lifestyle and systemic health management for these patients.

Our regression analysis further illuminated the specific factors that increase the likelihood of readmissions. Age brackets, discharge destinations, patient residence, and secondary diagnoses, such as deep venous thrombosis, emerged as significant contributors. These determinants could serve as a foundation for predictive models, aiding in the early identification and proactive management of high-risk patients.

Patients with PsA have a heightened burden of comorbidities, including cardiovascular diseases, metabolic disorders, and mental health issues [17]. These comorbidities not only compound the challenges faced by individuals with PsA but also contribute to the necessity of hospitalization. The intricate interplay between psoriasis and comorbidities is well documented. Psoriasis has been consistently linked to increased morbidity, and these comorbidities, in turn, are likely to exacerbate the outcomes of psoriasis. This bidirectional relationship underscores the importance of comprehensive care strategies that address both the primary condition and its associated health issues to improve the overall well-being of patients with PsA. The association between hospitalization and disease severity in patients with PsA is well established. Hospitalization often signifies uncontrolled symptoms, disease flares, and the need for intensive care, imposing substantial economic and personal costs. Recent evidence highlights the economic implications of readmissions [18,19]. Patients who are readmitted within 30 days face increased healthcare costs and morbidity risks, making the implementation of strategies to reduce readmissions in patients with PsA critical. Multidisciplinary care teams that include rheumatologists, dermatologists, and other specialists are essential for comprehensive disease management [20]. Early intervention during outpatient visits can help prevent disease flares that lead to hospitalization. Moreover, patient education and self-management programs are pivotal in empowering individuals to recognize and manage PsA symptoms, potentially averting the need for readmission.

The recurrence of hospital admissions within 30 days underscores potential gaps in the continuum of care for patients with PsA. These gaps can occur at various stages of the care journey, from initial diagnosis and treatment planning to post-discharge follow-up. Addressing these gaps requires a multifaceted approach. Enhanced care coordination among primary care providers, specialists, and allied health professionals can streamline care delivery. Regular communication and the use of electronic health records facilitate the sharing of patient information, ensuring that transitions between care settings are smooth and informed [21]. Implementation of transitional care programs, especially for high-risk patients with PsA, can bridge the gap between the hospital and home. These programs provide personalized care plans, medication reconciliation, and close monitoring, thereby reducing the risk of post-discharge complications. Equipping patients with PsA with knowledge and self-management skills is pivotal. Patient education initiatives should cover topics such as recognizing early signs of disease exacerbation, medication adherence, and lifestyle modifications to promote disease control.

The heightened comorbidity burden among patients with PsA, as observed in our study, necessitates a comprehensive approach to care. Current evidence suggests that effectively managing comorbid conditions enhances overall well-being and reduces hospital readmissions. For instance, addressing cardiovascular risk factors, such as hypertension and hyperlipidemia, is crucial given the increased cardiovascular morbidity and mortality observed in patients with PsA [22,23]. Comprehensive care plans should include regular monitoring, lifestyle modifications, and pharmacological interventions to mitigate these risks. Furthermore, mental health comorbidities, such as depression and anxiety, are prevalent in patients with PsA and can significantly impact disease management. Integrating mental health services into the care continuum can improve patient outcomes and potentially reduce hospitalizations.

Disparities in insurance coverage observed in our study highlight the need for healthcare policymakers to ensure equitable access to services for PsA patients. Several evidence-based strategies can address these disparities. Telehealth can improve access to care, particularly for individuals in underserved or remote areas. Expanding telehealth services and addressing reimbursement policies can enhance access to PsA specialists [24]. Implementing health literacy programs can further empower patients to navigate the healthcare system effectively, thereby reducing barriers to access. Outreach programs that raise awareness of PsA and available healthcare resources can help individuals overcome obstacles in accessing care. The prolonged LOS observed in readmissions signifies the complexity of cases requiring repeat hospitalization. Strategies to reduce LOS should include early intervention to prevent disease flares, optimized care coordination, and patient education to facilitate self-management and early recognition of symptoms. Furthermore, the substantial difference in total hospital charges between index hospitalizations and readmissions underscores the financial impact of PsA. Value-based care models that prioritize cost-effective interventions and reduce unnecessary hospitalizations can mitigate this financial burden [25,26].

Identifying predictors associated with an increased likelihood of readmission is important for each patient encounter. Evidence-based strategies should be developed to address these predictors. Younger age (18 to 40 years) is associated with an increased likelihood of readmission, indicating that tailored interventions should focus on the unique needs of younger patients with PsA. This may include psychosocial support, adherence counseling, and educational programs tailored to this age group. Patients discharged to home healthcare have a higher likelihood of readmission based on the findings of this study. Optimizing HHC services, ensuring comprehensive care plans, and close monitoring during home-based care can improve outcomes and reduce readmissions. Residence in the same state as the hospital is associated with an increased likelihood of readmission. This finding underscores the importance of post-discharge care. Implementing effective care transitions, including communication with local healthcare providers, can help mitigate readmission risks. Finally, strategies to reduce the risk of deep venous thrombosis, such as prophylactic measures and early detection, should be integrated into screening and care protocols for patients with PsA.

Strengths and limitations

This study possesses several distinctive strengths. It draws its study population from the largest hospital-based multipayer registry, imparting a robust foundation to our findings. It takes a comprehensive and thorough approach to assess the impact of PsA and its subsequent readmissions on the U.S. healthcare system. This in-depth analysis provides valuable insights into the severity of PsA-related readmissions, shedding light on a previously underexplored aspect of healthcare. However, as with all studies, certain limitations were inherent to our study. First, the NRD database lacks essential variables such as race and medication adherence, which could influence readmission rates. The retrospective nature of NRD introduces inherent difficulties with randomization and adjustment for residual confounding variables. In addition, the database presents hospitalization data without individual patient identifiers, which could result in an overrepresentation of cases involving multiple readmissions. Furthermore, the reliance on ICD-10 codes rather than clinical diagnosis within the NRD introduces the potential for coding errors. Despite these constraints, the analytical methods employed in our study, coupled with the substantial sample size, significantly advance our understanding of a relatively underexplored subject matter. Furthermore, our findings stimulate important discussions and set the stage for future controlled, multicenter prospective investigations into this important clinical topic.

## Conclusions

About one in three patients hospitalized with PsA are readmitted within 30 days of discharge. Patients with PsA who are readmitted have a higher burden of comorbidities, including cardiovascular, inflammatory, metabolic, and mental health disorders. Among these, acute kidney failure, depression, cholelithiasis, cholecystitis, and heart failure are the most common causes of readmission. Age <40 years, discharge to home healthcare, residence in the same state as the hospital, and a secondary diagnosis of inflammatory bowel disease or deep venous thrombosis were significantly correlated with an increased likelihood of readmission.
